# The Assessment of Carbon Dioxide Automated Angiography in Type II Endoleaks Detection: Comparison with Contrast-Enhanced Ultrasound

**DOI:** 10.1155/2018/7647165

**Published:** 2018-03-26

**Authors:** Chiara Mascoli, Gianluca Faggioli, Enrico Gallitto, Vincenzo Vento, Giuseppe Indelicato, Rodolfo Pini, Andrea Vacirca, Andrea Stella, Mauro Gargiulo

**Affiliations:** Vascular Surgery, DIMES, University of Bologna, Policlinico S. Orsola-Malpighi, Bologna, Italy

## Abstract

**Introduction:**

Iodinated contrast media completion angiography (ICM-A) may underestimate the presence of type II endoleak (ELII) after endovascular aortic repair (EVAR), particularly if they are at low flow. Contrast-enhanced ultrasound (CEUS) has been proposed as the gold standard in ELII detection during EVAR follow-up. Intraprocedural carbon dioxide (CO_2_) angiography has been shown to be useful in this setting; however no comparative studies including these three techniques are currently available. Our aim was to investigate the accuracy of a new automated CO_2_ angiographic (CO_2_-A) system in the detection of ELII, by comparing it with ICM-A and CEUS.

**Methods:**

A series of consecutive patients undergoing EVAR for abdominal aortic aneurysm (AAA) were enrolled and submitted to ICM-A and CO_2_-A during the procedure. The iodinated contrast media were delivered through an automatic injector connected to a pigtail catheter in the suprarenal aorta. CO_2_ was delivered through a recently available automatic injector connected to a 10 F sheath positioned in the external iliac artery. All patients were blindly evaluated by CEUS within postoperative day 1. The ICM-A and CO_2_-A ability to detect ELII was compared with that of CEUS through Cohen's concordance Index (*K*).

**Results:**

Twenty-one patients were enrolled in the study. One (5%), seven (33%), and four (19%) ELII were detected by ICM-A, CO_2_-A, and CEUS, respectively. The only ELII detected by ICM-A was also detected by CO_2_-A and CEUS. Three cases of ELII detected by CO_2_-A were not detected by CEUS. All ELII detected by CEUS were visualized by CO_2_-A. CEUS and ICM-A showed a poor agreement (Cohen's *K*: 0.35) while CEUS and CO_2_-A showed a substantial agreement (Cohen's *K*: 0.65) for ELII detection.

**Conclusion:**

CO_2_-A is safe and effective method for ELII detection in EVAR, with a significantly higher agreement with CEUS if compared with ICM-A. This trial is registered with 155/2015/U/Oss.

## 1. Introduction

Endovascular abdominal aortic repair (EVAR) has become widely accepted as a treatment of choice for abdominal aortic aneurysm repair, due to its minor invasiveness and lower short-term morbidity and mortality, if compared with open repair (OR) [1, 2].

The administration of iodinated contrast medium (ICM) is however necessary for adequate EVAR planning, procedure, and follow-up, and this can lead to progressive renal impairment especially in patients with a preexisting renal failure [3, 4].

The use of carbon dioxide (CO_2_) digital subtraction angiography (CO_2_-A) has been studied extensively [5–10] as an alternative contrast media in order to minimize the use of ICM during EVAR, especially in patients with severe renal insufficiency.

Many studies have compared CO_2_-A with ICM-A for their ability to visualize renal and hypogastric arteries showing good results [5] but the intraoperative endoleaks detection by CO_2_-A is still controversial [6, 7, 11, 12].

In particular, some studies have shown that CO_2_-A has good sensitivity and specificity for the evaluation of type I and III endoleaks but it is not a reliable method to detect type II endoleaks (ELII) [7, 12].

As a matter of fact, the presence of endoleaks after endovascular aortic repair (EVAR) can be investigated with different methods.

Contrast-enhanced ultrasound (CEUS) has high sensitivity and specificity for endoleaks, particularly if they are at low flow, and has been proposed as the gold standard during EVAR follow-up [13–15].

The aim of our study is to investigate the accuracy of a new automated CO_2_ angiographic system in the detection of endoleaks with particular attention to type II endoleak, by comparing it with ICM-A and CEUS.

## 2. Methods

### 2.1. Study Design

We performed a prospective single institution study between August and September 2016. The research protocol was approved and reviewed by the local Review Board.

All consecutive patients who underwent EVAR for infrarenal AAA were enrolled.

Preoperative intraoperative and postoperative data were collected and analysed, after obtaining patient informed consent.

All procedures were performed using two angiographic methods: automated conventional ICM angiography (ICM-A) and CO_2_ automated angiography (CO_2_-A), in a Philips hybrid operating theatre (https://www.philips.it/healthcare).

All patients were blindly evaluated for the presence of endoleaks type I/III and type II by CEUS within postoperative day 1.

The ICM and CO_2_ ability to detect endoleaks at completion angiography was compared with that of CEUS.

All patients were asked to start a low fiber diet 2 days before the endovascular repair and to take activated carbon in order to relieve intestinal gas and reduce the artefacts at the completion angiography as well as at the postoperative CEUS.

### 2.2. Procedures and Angiographic Methods

All the procedures were performed in a Philips hybrid operating theatre and all angiograms were obtained using the Integris Allura 12 DSA (https://www.philips.it/healthcare). EVAR was always performed through femoral surgical cutdown under spinal or general anaesthesia.

Patients considered at high risk for persistent type II endoleak (ELII) according to their anatomical characteristics (≥6 efferent patent vessels from AAA-sac, volume of AAA-sac intraluminal thrombosis <40%) [16] underwent intraoperative AAA-sac embolization as reported in a previous paper [17].

A diagnostic evaluation before and after the deployment of the endograft was performed in each procedure by injecting separately both ICM and CO_2_, in order to visualize the renal and hypogastric arteries. Each angiography was performed maintaining the blood pressure between 100 and 120 mmHg.

Iodinated contrast media were delivered through an automated injector (Medrad® Mark 7 Arterion® Injection System) connected to a pigtail catheter (5 F/65 mm length) placed in the suprarenal aorta, with an injection volume of 10 ml at a rate of 14 mL/s.

**Figure 1:**
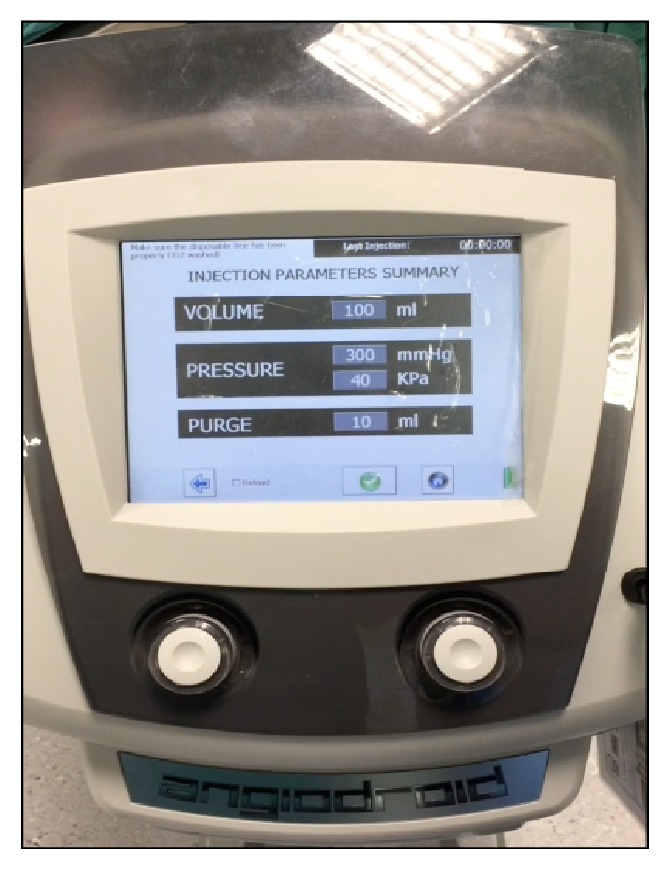
Angiodroid injection system that shows CO_2_ injection volume and pressure on the display.

Carbon dioxide was delivered through a recently available automatic injector system, Angiodroid (Angiodroid SRL, San Lazzaro, Bologna, Italy) (Figure 1), connected to the sidearm of 10 F/11 mm length sheath introducer in the external iliac artery, contralateral to the access of the main body. Before each injection the patient was placed in the Trendelenburg position with the feet elevated 10° degrees. Ten millilitres of CO_2_ was infused to fill the tubing with gas and eliminate the air, and after that, by apposite manipulation of the stopcocks, the sheath was back-bled through its sidearm and CO_2_ infused, creating a blood-CO_2_ interface with no air in the system. Subsequently 100 ml of CO_2_ was injected at a pressure of 300 mmHg.

Completion angiography was performed in order to evaluate the correct position of the endograft and the presence of endoleaks. The completion angiography was performed with both contrast media, in anterior-posterior positions, 45° LAO and RAO.

### 2.3. Contrast-Enhanced Ultrasound

All patients were blindly evaluated by CEUS on postoperative day 1.

All ultrasound (US) examinations, including baseline US, Doppler US, and CEUS, were performed with the same instrument (MyLab Twice eHD CrystaLine, CnTI software; Esaote SpA, Genova, Italy) and a multifrequency matrix array convex probe with a frequency range of 8.0–1.0 MHz (CA541; Esaote SpA) was used.

A sulfur hexafluoride-filled microbubble contrast agent (SonoVue; BR1, Bracco) was used for contrast examinations. All examinations were performed by one investigator (CM) who had great experience in contrast ultrasound and who was blinded to the results of the completion angiography.

The US examination started with B-mode evaluation of the aorta by live *x*-plane imaging where the maximal aneurysm diameter and the stent-graft were evaluated. The abdominal aorta was scanned from the diaphragm to the iliac arteries and the entire sac was analysed to detect possible colour flow within the aneurysm sac.

In the CEUS mode, the unenhanced and enhanced images were displayed simultaneously on the same screen (side-by-side technique) to identify the aorta and the collaterals previously evaluated with B-mode and Doppler US. SonoVue (Bracco) was injected into the antecubital vein as a 2.4 mL bolus (within 1 e 2 seconds), followed by a flush of 10 mL normal saline. The timer was activated promptly from the beginning of injection. The aorta was observed for at least 2 minutes until the signals from the microbubbles in the aorta disappeared, usually 5-6 minutes after the injection of the bolus. The whole process of CEUS was stored, for further analysis, on the hard disk incorporated in the machine [18].

### 2.4. Endoleak Detection

All patients were evaluated for the presence of endoleaks.

Endoleaks were defined according to White and May classification [19].

Intraoperative endoleaks were defined as “high-flow endoleaks” (type I/III endoleaks) if they appeared in the AAA-sac simultaneously to the presence of contrast media in the main body and “low-flow endoleak” (type II endoleak) if they appeared with some delay after the contrast media in the main body of the endograft.

Similarly, the endoleaks evaluation by CEUS was defined by monitoring the time of appearance contrast enhancement within the AAA-sac (if synchronous or delayed with respect to endograft enhancement) and site of appearance contrast enhancement.

Type I and III endoleaks were defined as contrast enhancement into the residual sac synchronous to endograft enhancement and that comes from the proximal or distal sealing zone (ELI) or from the endograft (ELIII) with centrifugal flow.

Type II endoleak was defined as a contrast enhancement into the residual sac appearing with ≥5 seconds' delay from endograft filling, starting either anteriorly or posteriorly in the AAA-sac with centripetal flow.

### 2.5. Endpoints and Definition

The primary endpoint was to evaluate the accuracy of a new automated CO_2_-A system in the detection of endoleaks by comparing it with ICM and CEUS.

A secondary endpoint was to determinate automated CO_2_-A system and ICM sensitivity and specificity in high-flow and low-flow endoleaks detection, in comparison with CEUS, considered as the gold standard.

### 2.6. Statistical Analysis

Continuous data are presented as mean and standard deviation (DS). Categorical data are given as counts and percentage. Differences in categorical and continuous variables between the two groups were analysed using, respectively, *χ*^2^ test (or Fisher exact test when appropriate) and Student's *t*-test.

The CEUS finding of any type of endoleaks was used as the criterion standard and the CEUS diagnoses were used to calculate statistical measures. The true positives, true negatives, false positives, and false negatives of ICM-A and CO_2_-A were calculated for detection of EL in the EVAR procedure. The sensitivity, specificity, positive predictive value (PPV), and negative predictive value (NPV) of ICM-A and CO_2_-A were calculated.

The endoleak's detection and identification were compared between ICM-A and CEUS and between CO_2_-A and CEUS. The Cohen *k* statistic was performed to assess agreement between the two diagnostic methods [20] (Table 1). The *k* coefficients were calculated for the detection of endoleaks. Statistical analysis was performed using SPSS 21.0 software (SPSS Inc., Chicago, Ill).

## 3. Results

### 3.1. Patients

Twenty-one consecutive patients were included into the study between August and September 2016.

The average patients' age was 76 ± 7 years; they were all men and the mean AAA size was of 57 ± 8 mm. The preoperative demographics and risk factors were reported in Table 2.

The procedure was performed under general (8 cases, 38.1%) or locoregional anaesthesia in (13 cases, 61.9%), respectively. Suprarenal (11 cases, 52.4%) and infrarenal (10 cases, 47.6%) fixation endografts were used according to the aneurysm anatomy.

The endograft was deployed in the intended location in 100% of cases. In 10 (47.6%) patients, considered at high risk of ELII due to the presence of the morphological risk factors cited before [16], a 45 cm long 5 F Terumo® Destination sheath was introduced over the wire, parallel to the contralateral limb and advanced under fluoroscopy to the AAA-sac. Once the endograft was completely deployed and the aneurysm excluded, Cook MReye coils (MReye Embolization Coil, IMWCE-38-16-45; Cook Medical, Limerick, Ireland),were advanced into the sac through 5 F sheath and intraoperative AAA-sac embolization was performed [17].

The median fluoroscopy time was 19.3 ± 5.3 minutes and the overall median procedure time was 173.3 ± 33.1 minutes. All intraoperative data were summarised in Table 3.

There were no intra- or perioperative major complications related to ICM-A or CO_2_-A; however 2 patients experienced an episode of severe hypotension during the procedure. They were both under locoregional anaesthesia and developed nausea and vomiting just before the onset of the hypotension. No evidence of either arterial or endograft defects was evident at intraoperative angiogram and immediate postoperative CT scan. Both episodes recovered promptly without any further sequelae. No other side effects were observed.

The mean time of hospital stay was 3.9 ± 1.2 days.

### 3.2. Endpoints

**Figure 2:**
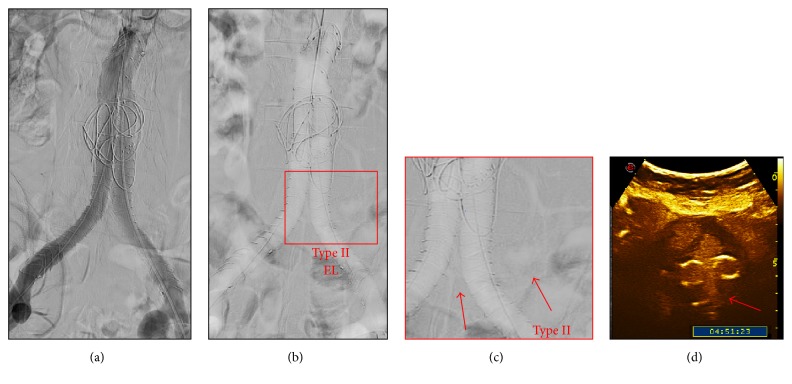
(a) Iodinated contrast media completion angiography that shows the good positioning of the infrarenal fixation endograft and the absence of endoleaks. (b) Carbon dioxide completion angiography that shows the good positioning of the infrarenal fixation endograft and the presence of ELII. (c) Magnification of ELII from sacral artery (red arrows indicates ELII coming from sacral artery). (d) Contrast-enhanced ultrasound that shows the presence of ELII with the inflow from sacral artery (red arrow).

**Figure 3:**
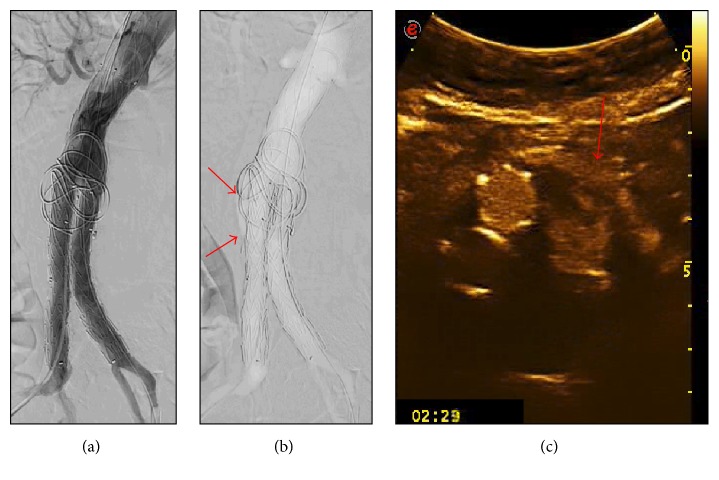
(a) Iodinate contrast media completion angiography that shows the good positioning of the suprarenal fixation endograft and the absence of endoleaks. (b) Carbon dioxide completion angiography that shows the good positioning of the suprarenal fixation endograft and the presence of ELII (as indicated by the red arrows). (c) Contrast-enhanced ultrasound that shows the presence of ELII with the inflow from inferior mesenteric artery (as indicated by the red arrow).

Completion angiography identified no high-flow endoleaks (ELI/III). One (5%) and seven (33%) low-flow-endoleaks (ELII) were detected by ICM-A and CO_2_-A (Figures 2(a), 2(b), 2(c), 3(a), and 3(b)), respectively. CEUS identified no ELI/III and 4 ELII were detected (Figures 2(d) and 3(c)). The only ELII detected by ICM-A was also detected by CO_2_-A and CEUS. This patient did not have the ELII at the CEUS performed at 6 and 12 months and had AAA-sac shrinkage of 5 mm at 12 months.

Three ELII detected by CO_2_-A were not detected by CEUS. No cases of ELII undetected by CO_2_-A were visualized by CEUS. ELII detection by CEUS, ICM-A, and CO_2_-A is summarised in Table 4.

A perfect agreement between CEUS and both ICM-A and CO_2_-A was observed for type I/III endoleak (Cohen's *K*: 1).

CEUS and ICM-A showed a poor agreement for ELII detection (Cohen's *K*: 0.35). A substantial agreement was observed between CEUS and CO_2_-A for ELII (Cohen's *K*: 0.65).

Carbon dioxide automated angiography and ICM-A sensitivity, specificity, PPV, and NPV for detection of high-flow and low-flow endoleaks are summarised in Tables 5 and 6, respectively.

## 4. Discussion

Our preliminary experience on 21 patients undergoing standard EVAR with a new standardized CO_2_ automated injection method is encouraging and particularly significant in terms of endoleak detection.

According to several studies, CO_2_ is safe and useful due to its physical and chemical properties, and its role as an effective contrast agent has already been validated. As a matter of fact, CO_2_ is a nonnephrotoxic, nonallergenic gas and its beneficial effect on preservation of renal function makes it a potential substitute for iodinated contrast media in EVAR procedures [6, 8, 11].

Despite the well-known advantages of CO_2_, its low-density and compressibility can cause some problems during its injection and this problem has limited its applicability.

Unlike other experiences in literature, in our series we have found CO_2_ effective in detecting endoleaks during EVAR, since our automated system allows a calculated and controlled injection in terms of dose and delivery rate, excluding the possibility of air contamination.

To the best of our knowledge, this is the first experience in the literature that analysed sensitivity and specificity of this new standardized CO_2_ automated angiographic system in endoleaks detection during EVAR procedure.

Type I/III endoleaks were always detected, independently from the contrast medium used, that is, at CO_2_-A and ICM-A. In previous experiences in literature [5, 7, 12] CO_2_-A was shown to have high sensitivity and specificity for high-flow endoleaks. In this study, we had no cases of ELI/III with all diagnostic methods. We can therefore assume that there was high concordance among the three diagnostic methods for the absence of ELI/III.

A real advantage of this new system seems to exist in EII detection where CO_2_-A showed to be more sensitive compared with ICM-A. While CO_2_-A allowed us to detect 7 ELII, only one of them was seen at ICM-A. In four cases the ELII was also confirmed by CEUS, resulting in a substantial agreement (Cohen's *K*: 0.65) between CEUS and CO_2_ but not between ICM-A and CO_2_ (Cohen's *K*: 0.35) with the criterion standard. This result has been never reported before.

The diagnostic accuracy of CO_2_ in ELII detection is debated in the literature. Chao et al. [6] in their experience on 16 patients with CO_2_ EVAR reported that CO_2_ was more sensitive in intraoperative endoleaks detection compared with ICM and they supposed that this was determined by the CO_2_ lower viscosity. Huang et al. [7] showed that, despite acceptable sensitivity and specificity in detecting type I endoleak, CO_2_-A has poor sensitivity and poor positive predictive value in the detection of ELII. Sueyoshi et al. [12], in their study on 40 patients undergoing EVAR by both ICM-A and CO_2_-A, reported poorer sensitivity in ELII detection by CO_2_, but those ELII detected by CO_2_ tended to persist over 6 months. Therefore, the authors concluded that CO_2_-A was a reliable tool for the detection of persistent ELII. The authors speculated that the posterior location of the lumbar arteries as well as the volume and speed of blood flow may contribute to ELII CO_2_-A visualization. The sensitivity (1.00) and specificity (0.82) for low-flow endoleaks detection using CO_2_-A in our study was higher than other experiences of the literature [7, 12], with a significant advantage compared with ICM. The reason for that may be in the automated delivery system, which reduces the possible variability of infusion pressure of manual injection. Further studies are however needed in order to support this speculation.

The high sensitivity of CO_2_ for low-flow endoleaks could have some important clinical implication. First, it could limit the use of conventional contrast media to the diagnostic angiography only, performing the completion angiography only with CO_2_. Second, it could be useful during the procedure of intraoperative AAA-sac embolization [17] as a method to confirm the AAA-sac thrombosis.

Finally, ELII are visualized faster by CO_2_-A than by ICM-A due to the lower viscosity [11] and this could reduce the radiation time exposure for both patient and operator.

In our study, we reported three cases of endoleaks detected by CO_2_-A and undetected by CEUS. We interpreted them as very low-flow ELII, sealed within the first postoperative day, before the evaluation by CEUS. Another interpretation could be that these cases were type IV endoleaks, visible thanks to the lower viscosity of CO_2_ if compared with ICM and blood.

Two cases of possible adverse events related to CO_2_ infusion have been observed in this study. The severe hypotension, which occurred (systolic pressure < 60 mmHg) during EVAR procedure, was preceded by nausea and vomiting, resolved spontaneously in both cases, and was similar to other side effects of CO_2_ injection reported by others [6].

This study has some limitations. First of all, this is a preliminary experience with a standardized technique of CO_2_ automated injection and the learning curve should therefore be considered. Next, this was an observational, prospective study with a small sample size; additional studies involving a larger number of patients will be needed in order to improve the set injection parameters and to validate the diagnostic accuracy in endoleaks detection.

## 5. Conclusion

Carbon dioxide automated angiography using this new automated system is a safe and effective method for endoleak detection in EVAR. In this series CO_2_-A showed high sensitivity and specificity for high-flow and low-flow endoleaks and higher agreement with CEUS if compared with ICM-A for type II endoleak detection.

## Figures and Tables

**Table 1:** Interpretation for the *Cohen k coefficient*.

*Cohen k coefficient*	Agreement
0	Absent
0.01–0.20	Slight
0.21–0.40	Poor
0.41–0.60	Moderate
0.61–0.80	Substantial
0.81–0.99	Almost perfect
1	Perfect

**Table 2:** Demographic and clinical characteristics.

	*N*	%
Demographic		
Age (yrs)	76.1	6.7^*∗*^
Sex (male)	21	100

Clinical		
HPT	20	95.2
COPD	7	33.3
CAD	4	19.0
Atrial fibrillation	3	14.3
Dyslipidemia	13	61.9
CRF (GFR < 60 mL/min/1.73 m^2^)	7	33.3
Dialysis	2	9.5
Smoke	15	71.4
Obesity	3	14.3
Peripheral artery disease	2	9.5

^*∗*^Mean and standard deviation (SD); data are expressed as mean and standard deviation (SD) or number and percentage. HPT: hypertension; COPD: chronic obstructive pulmonary disease; CAD: coronary artery disease; CRF: chronic renal failure expressed as glomerular filtration rate (GFR) < 60 mL/min/1.73 m^2^.

**Table 3:** Intraoperative data.

Intraoperative data	*N*	%
Procedure time (m)^*∗*^	173.3	(33.1)
Endograft		
Suprarenal fixation	11	52.4
Infrarenal fixation	10	47.6
Fluoroscopy time (m)^*∗*^	19.3	(5.3)
DAP (mGy*∗*m^2^)^*∗*^	19.0	(16.7)
General anesthesia (n°)	8	38.1
Spinal anaesthesia (n°)	13	61.9
AAA-sac embolization	10	47.6

^*∗*^Mean and standard deviation (DS); DAP: dose-area product (mGy*∗*m^2^); AAA-sac embolization: abdominal aortic aneurysm intraoperative sac embolization.

**Table 4:** Type II endoleak detected by CO_2_-A, ICM-A, and CEUS.

ID	CO_2_-A	ICM-A	CEUS
# 5	✓	−	✓
# 9	✓	−	−
# 14	✓	−	✓
# 16	✓	−	−
# 17	✓	✓	✓
# 18	✓	−	−
# 21	✓	−	✓

ID: patient series number; ✓: type II endoleak presence; −: type II endoleak absence.

**Table 5:** Sensitivity, specificity, positive predictive value (PPV), and negative predictive value (NPV) of CO_2_-A for type II endoleak detection during EVAR.

Endoleak	Sensitivity	Specificity	PPV	NPV
Type I	1	1	1	1
Type II	1	0.82	0.57	1
Type III	1	1	1	1

**Table 6:** Sensitivity, specificity, positive predictive value (PPV), and negative predictive value (NPV) of ICM-A for type II endoleak detection during EVAR.

Endoleak	Sensitivity	Specificity	PPV	NPV
Type I	1	1	1	1
Type II	0.25	1	1	0.85
Type III	1	1	1	1
